# Efficient isolation of live microglia with preserved phenotypes from adult mouse brain

**DOI:** 10.1186/1742-2094-9-147

**Published:** 2012-06-28

**Authors:** Maria Nikodemova, Jyoti J Watters

**Affiliations:** 1 Department of Comparative Biosciences, University of Wisconsin, Madison, WI, 53706, USA

**Keywords:** TNF-α, Percoll, Sucrose, Anti-myelin beads, Immunomagnetic separation, Neuroinflammation, CD11b, Lipopolysaccharide

## Abstract

**Background:**

Microglial activation plays a key role in the neuroinflammation associated with virtually all CNS disorders, although their role in normal CNS physiology is becoming increasingly appreciated. Neuroinflammation is often assessed by analyzing pro-inflammatory mediators in CNS tissue homogenates, under the assumption that microglia are the main source of these molecules. However, other cell types in the CNS can also synthesize inflammatory molecules. Hence, to enable direct analysis of microglial activities *ex vivo,* an efficient, reliable, and reproducible method of microglial isolation is needed.

**Methods:**

After enzymatic digestion of brain tissues and myelin removal, CD11b^+^ cells were isolated using immunomagnetic separation, yielding highly purified microglia without astrocyte or neuronal contamination. We used three methods of myelin removal (30% Percoll, 0.9 mol/l sucrose and anti-myelin magnetic beads), and compared their effects on microglial viability and yield. To determine whether the isolation procedure itself activates microglia, we used flow cytometry to examine microglial properties in brain-tissue homogenates and isolated microglia from control and lipopolysaccharide (LPS) -treated mice.

**Results:**

This method yielded a highly purified CD11b^+^ cell population with properties that reflected their *in vivo* phenotype. The viability and yield of isolated cells were significantly affected by the myelin removal method. Although the microglial phenotype was comparable in all methods used, the highest viability and number of CD11b^+^ cells was obtained with Percoll. Microglia isolated from LPS-treated mice displayed a pro-inflammatory phenotype as determined by upregulated levels of TNF-α, whereas microglia isolated from control mice did not.

**Conclusions:**

Immunomagnetic separation is an efficient method to isolate microglia from the CNS, and is equally suitable for isolating quiescent and activated microglia. This technique allows evaluation of microglial activities *ex vivo*, which accurately reflects their activities *in vivo*. Microglia obtained by this method can be used for multiple downstream applications including qRT-PCR, ELISA, Western blotting, and flow cytometry to analyze microglial activities in any number of CNS pathologies or injuries.

## Introduction

Emerging evidence indicates the active involvement of microglia, CNS resident innate immune cells, in virtually all aspects of physiology in the healthy, diseased, and injured CNS [1-3]. Microglia play a key role in the neuroinflammation associated with many neurodegenerative, ischemic, and traumatic disorders [4-12]. The current methods used to study microglial cells have a number of limitations, thus there is a need for better tools enabling analysis of microglial properties in various physiological or pathological situations. Although immunohistochemistry is invaluable in assessing many microglial properties such as morphology, proliferation, site of activation and others, this method is not generally suitable for analyzing cytokine production, one of the key microglial responses to many extrinsic and intrinsic CNS insults.

Neuroinflammation is often assessed by analyzing cytokine expression in CNS tissue homogenates; however, this method lacks cell specificity even though microglia are usually presumed to be the source of inflammatory molecule production in these samples. Because astrocytes and even neurons can also exert some immune activities, such as production of cytokines [13-15], it is important to distinguish the cellular source of these pro-inflammatory mediators. Hence, an efficient, reliable and highly reproducible method of microglial isolation is needed to allow direct analysis of their properties *ex vivo,* regardless of their phenotype (e.g. activated or quiescent)*.*

Unlike astrocytes and neurons for which antibodies recognizing extracellular epitopes of cell type-specific membrane proteins are not available, the expression of CD11b on microglia allows efficient antibody-based separation of these cells from CNS tissues. In this study, we used immunomagnetic separation of CD11b^+^ cells, a technique that reliably yields highly purified microglia (without contamination by neuronal or glial cells) and preserves their phenotype during isolation. The method itself does not activate the isolated cells, and it is suitable for both quantitative and qualitative analysis of RNA and proteins in microglia. Although we have used this method in earlier studies [16,17], and magnetic cell separation has been previously used to isolate microglia from brain regions [18] and to separate them from astrocytes in primary culture [19], here we report a modified isolation method that does not require Percoll density gradient centrifugation. We also provide a basic characterization of the microglia isolated by this method, their viability based on three different methods of myelin removal, the effect of the isolation procedure itself on the microglial phenotype, and the efficiency with which microglia can be isolated. These parameters, which were not evaluated in previous studies, are critical for assessing the utility of this method for studying microglial contributions to neuroinflammation.

## Methods

### Animals

All experiments were conducted in AAALAC-accredited facilities under protocols approved by the University of Wisconsin Institutional Animal Care and Use Committee.

Male adult (2–4 month old) ICR/CD1 mice (Charles River, Wilmington, MA, USA) were housed under standard conditions (12 hours light/dark cycle, with water and food available *ad libitum*).

*LPS treatment -* Lipopolysaccharide (LPS; *E. coli* 011:B4, Sigma Chemical Co., Missouri, MO, USA) at a dose of 1 mg/kg body weight or vehicle (PBS; 100 μl) were administered to mice by intraperitoneal (i.p.) injection. Microglial cells were isolated from brains 20 hours post-injection.

*Macrophage isolation* - Macrophages were obtained by peritoneal lavage with 10 ml of cold HBSS.

### Microglial isolation

**Figure 1:**
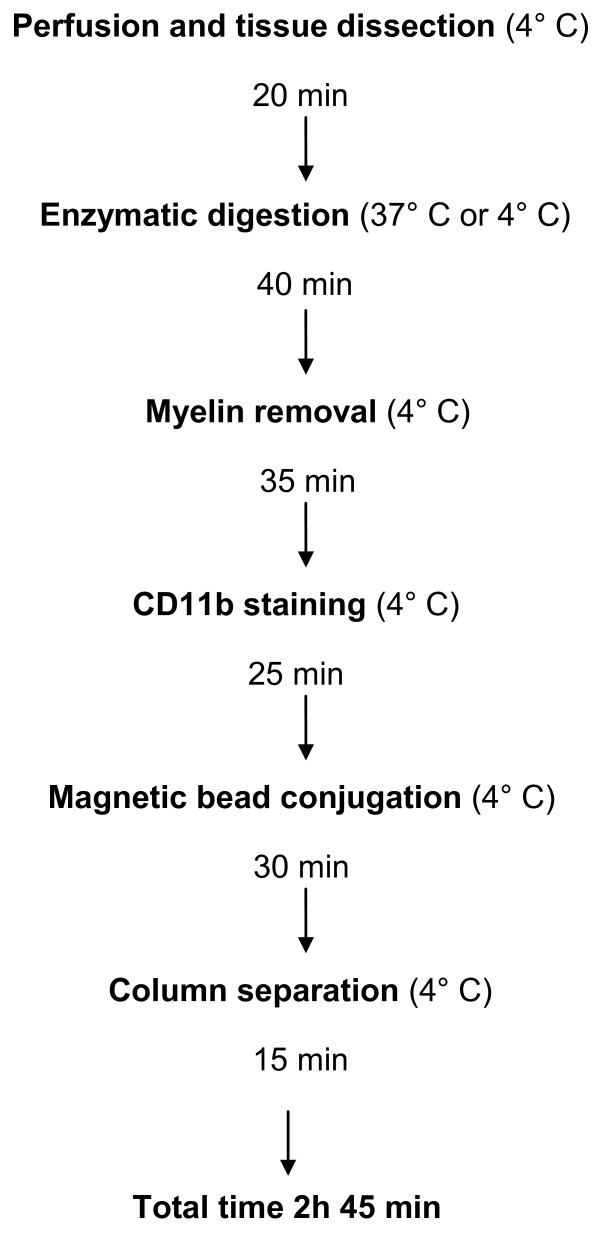
**Flow chart of CD11b**^**+**^**cell isolation.** Animals were perfused with ice-cold PBS to remove circulating immune cells. Brains were dissected and tissue was gently dissociated using enzymatic digestion. Myelin was removed using one of three different methods (Percoll or sucrose centrifugation, or myelin magnetic beads). Cells were stained with anti-CD11b antibodies and then incubated with magnetic beads. Samples were extensively washed on columns in a magnetic field to obtain the CD11b^+^ or effluent (CD11b^-^) cell fractions. The total time for isolation was less than 3 hours.

Microglial cells were isolated from brains as we described previously [17]. The overview of the method is depicted in Figure 1. Briefly, after perfusion with ice-cold PBS, brains were dissected, weighed, and enzymatically digested using Neural Tissue Dissociation Kit (Miltenyi Biotec, Germany) for 35 min at 37°C (if necessary, the digestion can be performed on ice, but this extends the digestion time). Further processing was performed at 4°C. Tissue debris was removed by passing the cell suspension through a 40 μm cell strainer. After myelin removal (see below), cells were stained with PE-conjugated anti-CD11b antibodies (Miltenyi Biotec, Germany) in IMAG buffer (PBS supplemented with 0.5% BSA and 2 mM EDTA) for 10 minutes followed by incubation for 15 minutes with anti-PE magnetic beads. CD11b^+^ cells were separated in a magnetic field using MS columns (Miltenyi Biotec, Germany). The amounts of antibodies and magnetic beads were calculated based on the number of cells obtained after myelin removal, using the manufacturer’s guidelines. Both the CD11b^+^ and CD11b^-^ (effluent) fractions were collected and used for further analyses.

### Myelin removal methods

#### Percoll

After enzymatic dissociation, cells were resuspended in 30% Percoll (GE Healthcare, Princeton, NJ, USA) and centrifuged for 10 minutes at 700 g. The supernatant containing the myelin was removed, and the pelleted cells were washed with HBSS, followed by immunomagnetic isolation as described above.

#### Sucrose

Dissociated cells were resuspended in 0.9 mol/l sucrose prepared in HBSS, and processed as described for the Percoll method.

#### Anti-myelin beads

Cells were resuspended in IMAG buffer supplemented with magnetic myelin removal beads (200 μl/brain; Miltenyi Biotec) and incubated for 15 minutes. Myelin was removed by magnetic separation using LS columns (Miltenyi Biotec), with three columns used per brain. The cells were collected and processed as described above.

### Cell viability

Isolated CD11b^+^ cells were stained with Trypan blue and the numbers of live and dead cells were counted under the microscope based on dye exclusion. Alternatively, cells were stained with Live/Dead fixable green Stain in accordance with the manufacturer’s protocol (Invitrogen, Carlsbad, CA, USA). This staining is compatible with subsequent cell fixation/permeabilization procedures and intracellular staining for flow cytometry analysis.

### Flow cytometry

CD11b^+^ cells and the effluent fractions (CD11b-negative cells) obtained from the isolation procedure were resuspended in IMAG buffer and immunostained with anti-CD45 antibodies (BD Pharmingen) for 10 minutes at 4°C. After washing, the cells were permeabilized and fixed (BD Cytofix/Cytoperm Solution; BD Biosciences) for 25 minutes at 4°C, followed by intracellular staining with antibodies for GFAP (Cell Signaling, Danvers, MA, USA) or NeuN (Millipore Billerica, MA, USA) in Perm/Wash buffer (BD Biosciences). GFAP and CD45 antibodies were fluorochrome-conjugated, and used at final dilutions of 1:100. For NeuN staining, secondary goat anti-mouse fluorochrome-conjugated antibodies were used at a dilution 1:1000 (Invitrogen, Carlsbad, CA, USA). Finally, the cells were fixed with 1.6% paraformaldehyde and stored at 4°C until analysis by flow cytometry (within 24 hours).

#### TNF-α immunostaining

Whole brain homogenates and isolated CD11b^+^ cells were obtained from control (PBS) mice or mice injected with LPS, as described above. Cells were resuspended in DMEM supplemented with 10% FBS, penicillin/streptomycin and 3 μl/ml of GolgiPlug (BD Biosciences), and incubated at 37°C for 4 hours. The cells were then processed for intracellular staining as described above with APC-conjugated TNF-α antibodies (1:100 dilution; BD Pharmingen).

### RNA isolation and qRT- PCR

RNA isolation and quantitative reverse transcriptase (qRT)-PCR was performed as we described in detail previously [16,17]. Briefly, cells were resuspended in Tri-Reagent (Sigma, MO, USA) and total RNA was isolated in accordance with the manufacturer’s protocol. MMLV reverse transcriptase (Invitrogen) was used to synthesize cDNA from 0.5 μg of total RNA. Quantitative PCR was performed using Power SYBR Green solution (Applied Biosystems, Foster City, CA, USA) with gene expression normalized to 18 S. Primer sequences are provided in Table 1. Relative expression levels were determined by the ΔΔCt method, and the data are expressed as the fold change relative to the sample indicated in each figure. Gene expression was considered undetectable if the Ct value was greater than 35 cycles.

**Table 1:** Primer sequences

	**Direction**	**Sequence 5′→3′**
18 S	Forward	CGGGTGCTCTTAGCTGAGTGTCCCG
	Reverse	CTCGGGCCTGCTTTGAACAC
CD11b	Forward	AAGGATTCAGCAAGCCAGAA
	Reverse	GGAGGGATGAGAGTCCACAT
GFAP	Forward	TGCTGGAGGGCGAAGAAAACCG
	Reverse	TTTGGTGCTTTTGCCCCCTCGG
NeuN	Forward	GTTGCCTACCGGGGTGCACAC
	Reverse	TGCTCCAGTGCCGCTCCATAAG
TNF-α	Forward	TGTAGCCCACGTCGTAGCAA
	Reverse	AGGTACAACCCATCGGCTGG

### Statistical analysis

Each experiment was performed independently at least three times. Results are expressed as the mean ± SE. The significance of the difference between means was assessed by a Student’s *t*-test, and *P* < 0.05 was considered significant. Statistical analyses were performed using SigmaStat software (Systat, San Jose, CA, USA).

## Results

### Viability and yield of CD11b^+^ cells

**Figure 2:**
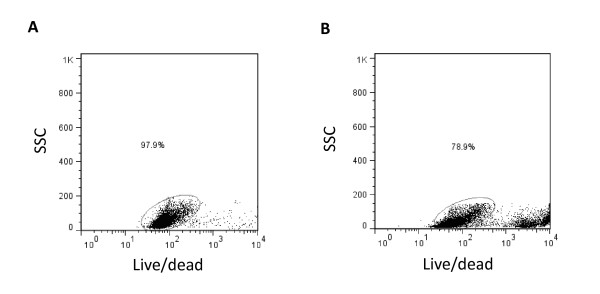
**Yield and survival of isolated CD11b**^**+**^**cells.** Brain tissues were enzymatically digested followed by centrifugation in **(A)** 30% Percoll or **(B)** 0.9 mol/l sucrose to remove myelin. After immunomagnetic separation of the CD11b^+^ cell population, cells were stained with Live/Dead fixable green stain, and the proportion of surviving cells was determined by flow cytometry.

Because myelin can interfere with downstream applications such as immunomagnetic cell separation or flow cytometry, its removal is recommended for the adult rodent CNS. We found that cell viability and yield depended on the myelin removal method (Table 2). We tested three different methods for myelin removal: centrifugation in either 30% Percoll or 0.9 mol/l sucrose, or myelin removal magnetic beads with subsequent column separation in a magnetic field. We found that cell viability was highest using Percoll, followed by anti-myelin beads, as determined by either Trypan blue exclusion (Table 2) or cell staining with the Live/Dead stain (Figure 2). The number of recovered viable cells using the Percoll method was almost twice that of the sucrose method (Table 2). The proportion of viable cells assessed by Live/Dead staining (Figure 2) appears to be higher than with Trypan blue exclusion (Table 2). This is likely due to the difficulty in distinguishing cell debris from dead cells under the microscope; cell debris can be gated out in flow cytometry analysis based on forward and side scatter parameters.

**Table 2:** **Comparison of the number of isolated live CD11b**^**+**^**cells determined by Trypan blue exclusion (n = 3)**

	**Live cells (%)**	**Live cells/mg tissue**	**Live cells/brain**
Percoll	93 ± 0.9	2011 ± 167	994,000 ± 82,454
Anti-myelin beads	81 ± 1.8	1792 ± 241	878,400 ± 110,963
Sucrose	61 ± 4.4	884 ± 88	409,791 ± 53,427

### Analysis of the CD11b^+^ fraction

**Figure 3:**
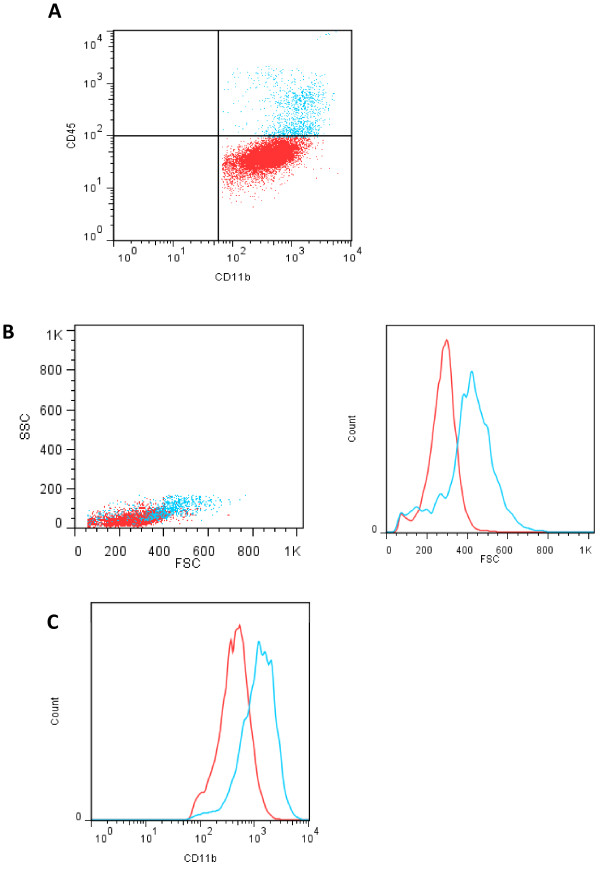
**Analysis of the CD11b**^**+**^**fraction. (A**) After isolation, cells in the CD11b^+^ fraction were stained with anti-CD45 antibodies and analyzed by flow cytometry. Two CD45-expressing populations were identified: low (red) and high (blue). **(B**) Forward scatter (FSC) and side scatter (SSC) characteristics of CD11b^+^/CD45^low^ (red) and CD11b^+^/CD45 ^high^ (blue) are shown. A histogram of FSC parameters comparing CD45^low^ and CD45^high^ cells is shown on the right. **(C)** Histogram showing the differences in CD11b expression between CD45^low^ and CD45^high^ cell populations.

The cells in the CD11b^+^ fraction were analyzed by flow cytometry and qRT-PCR for the expression of key markers distinguishing microglia, astrocytes, and neuronal cells. In the CD11b^+^ fraction, we found that less than 1% of the cells were GFAP^+^, and NeuN^+^ cells were undetectable, indicating high purity of CD11b^+^ cells isolated from brain tissue. We identified two CD11b^+^ cell populations in brain that differed in their expression levels of CD45 and CD11b, as assessed by flow cytometry (Figure 3A). Microglia, identified as CD11b^+^ and CD45^low^, comprised the highest proportion of cells (88–96%). We obtained similar results with all three methods of myelin removal. Further analysis of CD45^high^ and CD45^low^ cells revealed differences in their forward and side scatter properties (Figure 3B), suggesting that CD45^high^ cells are larger than CD45^low^ cells. In addition, CD45^high^ cells also expressed higher levels of CD11b (Figure 3C). These data suggest that CD45^low^ and CD45^high^ cells are distinct populations. However, it remains to be determined whether these populations are also functionally distinct, and how their relative proportions change in different physiological and pathological conditions.

**Figure 4:**
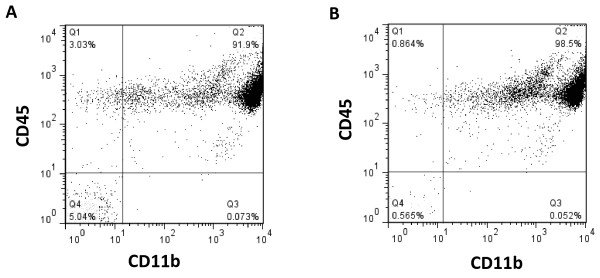
**Analysis of peritoneal macrophages. (A)** Peritoneal lavage samples containing abundant macrophages were subjected to immunomagnetic separation for CD11b^+^ cells (**B**). Cell populations expressing a range of CD11b levels were isolated with comparable efficiency. Both figures show the entire ungated cell population.

To test whether this method is suitable for isolation of cells with a wide range of CD11b expression levels, we subjected cells obtained by peritoneal lavage containing predominantly monocytes, macrophages, and dendritic cells, to the same immunomagnetic isolation procedure (Figure 4). The fluorescence intensity of CD11b protein levels in the peritoneal lavage spanned almost three orders of magnitude (Figure 4A). After immunomagnetic separation, there was complete recovery of CD11b^+^ cells suggesting that this method is suitable for isolating cells with a wide range of CD11b expression (Figure 4B).

**Figure 5:**
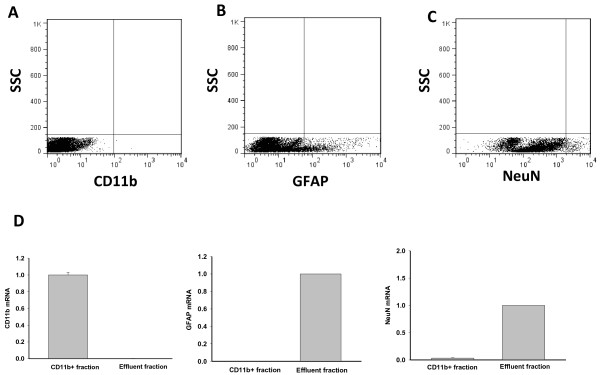
**Analysis of the CD11b**^**-**^**(effluent) fraction.** Cells in the effluent fraction were stained with anti-GFAP or anti-NeuN antibodies. **(A)** No CD11b^+^ cells were detected in this fraction, suggesting high efficiency of the CD11b^+^ cell separation from CNS tissues. As expected, this fraction consisted of large populations of **(B)** astrocytes and **(C)** neurons. **(D)** These observations were confirmed by mRNA analysis. High expression of CD11b was found only in the CD11b^+^ fraction, with undetectable levels in the effluent fraction, whereas high mRNA levels of GFAP and NeuN were found only in the effluent fraction.

Analysis of mRNA from the CD11b^+^ fraction obtained from brains showed high levels of CD11b expression and undetectable levels of GFAP and NeuN, which are markers of astrocytes and neurons, respectively (Figure 5D).

### Analysis of the CD11b^-^ fraction

Flow cytometry analysis confirmed the absence of CD11b^+^ cells in the effluent fraction, indicating high efficiency of CD11b^+^ cell separation from the cell suspension (Figure 5A). As expected, many GFAP^+^ and NeuN^+^ cells were detected in this fraction (Figures 5B, C). The analysis of mRNA by qRT-PCR confirmed high levels of NeuN and GFAP mRNA in the effluent fraction, and undetectable levels of CD11b mRNA (Figure 5D).

### Microglial phenotype

**Figure 6:**
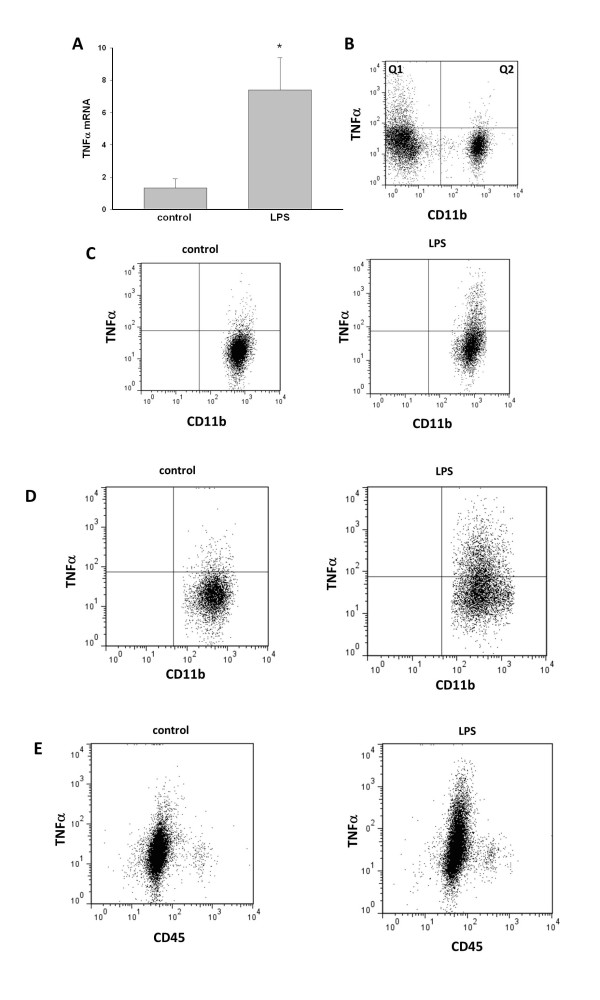
**Analysis of the microglial phenotype.** We compared the phenotype of microglia isolated from the brains of control and LPS-treated mice. **(A**) qRT- PCR identified significantly upregulated levels of TNF-α mRNA in CD11b^+^ cells isolated from LPS-treated mice. TNF-α protein content in CD11b^+^ cells was compared **(B, C)** before and **(D)** after the immunomagnetic isolation procedure. **(B**) In brain tissue homogenates from control mice, only a small percentage of microglia contained TNF-α (Q2), but there was a CD11b negative cell population with high TNF-α content (Q1). **(C**) TNF-α in brain tissue homogenates gated on CD11b^+^ cells. **(D**) The analysis of TNF-α in isolated microglial cells showed that the frequency of TNF-α-positive cells increased similarly (by 6-fold) in CD11b^+^ cells before and after isolation. **(E)** TNF-α was produced predominantly by CD11b^+^/CD45^low^ cells. The analysis of CD45 expression showed that there was not substantial infiltration of peripheral macrophages 20 hours after intraperitoneal injection of LPS, as we did not detect increased CD45 levels. *P < 0.05, n = 4.

To determine whether the isolation procedure itself alters microglial properties, and if microglia retain their *in vivo* phenotype during the isolation procedure, we compared the production of TNF-α in microglia isolated from control or LPS-treated mice. LPS is a well-established inducer of neuroinflammation and microglial activation. We found a 7-fold increase in microglial mRNA levels of this cytokine after LPS administration, consistent with a pro-inflammatory phenotype (Figure 6A).

This observation was also confirmed by flow cytometry analysis of TNF-α protein levels. We examined the production of TNF-α in microglial cells before and after their immunomagnetic isolation (by comparing TNF-α levels in CD11b^+^ cells from brain-tissue homogenates versus levels in isolated microglia), to assess the effects of the isolation procedure itself on this indicator of microglial activation. Although microglia in the normal, healthy CNS contained low levels of TNF-α, we found that there was a significant source of this protein in the normal CNS that did not originate from microglia (Figure 6B), as these cells were positive for TNF-α but negative for CD11b.

Microglia subjected to the isolation procedure from LPS-treated mice retained their pro-inflammatory phenotype, whereas microglia isolated from control mice maintained a quiescent phenotype as assessed by CD11b, CD45, and TNF-α protein levels. The increase in the frequency of TNF-α^+^/CD11b^+^ cells that was induced by LPS was similar (6-fold) before (Figure 6C) and after (Figure 6D) immunomagnetic separation, suggesting that the isolation procedure does not further activate them. Our results also showed that TNF-α was upregulated predominantly in CD11b^+^/CD45^low^ cells, suggesting that microglia were the primary responders to LPS challenge (Figure 6E). Interestingly, the relative proportions of CD11b^+^ cells that are either CD45^low^ or CD45^high^ also did not change after peripheral LPS administration, suggesting that there is no detectable infiltration of peripheral CD11b^+^ cells into the CNS at this time after intraperitoneal LPS injection. Importantly, similar observations were made regardless of the method used for myelin removal.

## Discussion

We describe here an efficient method that is suitable for the isolation of both quiescent and activated microglia from the CNS, allowing for the evaluation of microglial activities *ex vivo*, which accurately reflect their activities *in vivo*. This method is especially valuable for studying microglial properties in various CNS disorders; a better understanding of their role in pathological processes and the mechanisms that regulate their activities will enable the development of new strategies to therapeutically target microglia.

We identified two populations of CD11b^+^ cells in the healthy brain, which differ in their CD45 expression levels. Microglia that express very low CD45 levels comprised the largest population of CD11b^+^ cells in the CNS (about 88-96%). Macrophages and/or other myeloid cells were also present, but in much less abundance. However, it is likely that the proportions of these subpopulations will differ under various pathological conditions, and therefore it will be important to establish the abundance of each population during the CNS condition of interest before making judgments about microglial activities.

We used three different methods for myelin removal from the adult CNS, a step necessary for downstream applications such as immunostaining or flow cytometry. We found that all methods were very efficient at removing myelin, and none of them changed microglial properties. Several factors may be considered when deciding which method to choose for myelin removal. The most practical one is cost, with sucrose being the most cost-effective method of the three evaluated here. However, downstream applications should also be taken into account, especially when interested in quantifying changes in microglial cell numbers or in obtaining larger numbers of cells. In this case, Percoll is a cost-effective solution and likely the best option as cell yield and viability is much improved compared to sucrose.

Many pro-inflammatory cytokines, including TNF-α, are secreted from macrophages upon LPS stimulation by constitutive exocytosis. This process is dependent on receptor-mediated transcription; for example, through Toll-like receptor 4 after LPS administration [20,21]. In this secretory pathway, a newly synthesized protein is quickly trafficked in recycling endosomes via the Golgi complex to the membrane surface, where it is cleaved by TNF-α-converting enzyme, followed by the rapid release of the ectodomain as a soluble cytokine [21], a process that can take less than 20 minutes. Therefore, when analyzing protein levels of these cytokines, it is often necessary to use an inhibitor of the Golgi apparatus in order to accumulate proteins intracellularly and enable their detection. Indeed, when analyzing isolated microglial cells from LPS-treated animals without prior incubation in the presence of GolgiPlug, we were unable to detect TNF-α by flow cytometry in CD11b^+^ cells despite detection of strongly upregulated levels of TNF-α mRNA. Using the same method of microglial analysis *ex vivo*, we have previously reported strong upregulation of many inflammatory genes including TNF-α, IL-1β, IL-6 and IL-10 after LPS injection directly into the brain [17], as well as changes in purinergic receptor expression with age and sex [16]. Because of limitations with all currently available methods used in microglial research, we can only approximate the true microglial phenotype *in vivo*. However, the ability to capture differences in microglial properties under different conditions or treatments suggests that the method for microglial isolation described here is suitable to assess changes in microglial activities in many CNS pathologies.

In the present study, we found that there are significant levels of TNF-α that do not originate from microglia in the healthy CNS. Although it was beyond the scope of the present study to identify the cellular source or the biological effects of this non-microglial TNF-α, this is an important consideration when evaluating neuroinflammation based on the expression of pro-inflammatory cytokines in CNS homogenates composed of multiple cell types. Because microglial cells are among the least abundant populations in the CNS, subtle changes in microglial gene expression may not be detectable. Moreover, other cell types in the CNS may express pro-inflammatory cytokines or growth factors, as we show here; therefore, it is inappropriate to assume that expression levels of these molecules found in whole-tissue homogenates always reflects microglial activities.

Another advantage provided by this method is the ability to investigate immune responses, such as cytokine production, in CNS cell populations that are depleted of microglia and/or macrophages, an approach that is not possible when microglia are isolated using a Percoll gradient. Simultaneous examination of both the CD11b^−^ and CD11b^+^ fractions will provide important insights into neuroinflammatory responses in various pathological conditions, and distinguish responses originating in microglia from those of other CNS cell types.

## Conclusion

This method provides a highly efficient and powerful tool for investigating microglial properties *ex vivo*. Microglia can be isolated either from the whole CNS or from distinct CNS regions as small as the hippocampus. Based on the cell surface marker and inflammatory gene expression parameters we have assessed here, the method itself does not appear to activate the microglia, and the isolated cells retain properties very similar to those *in vivo,* making this method suitable for the assessment of multiple microglial parameters. The use of this technique will enable the specific evaluation of microglia in the context of neuroinflammation, instead of inferring their activities based on inflammatory gene expression from mixed-cell CNS homogenates.

## Abbreviations

BSA, bovine serum albumin; CNS, Central nervous system; DMEM, Dulbecco’s modified Eagle’s medium; EDTA, ethylene diamine tetraacetic acid; ELISA, enzyme-linked immunosorbent assay; FBS, Fetal bovine serum; GFAP, Glial fibrillary acidic protein; HBSS, Hank’s balanced salt solution; IL, Interleukin; LPS, Lipopolysaccharide; MMLV, Moloney murine leukemia virus; PBS, Phosphate-buffered saline; qRT-PCR, quantitative reverse transcriptase polymerase chain reaction; TNF, Tumor necrosis factor..

## Competing interests

The authors declare no conflict of interest.

## Authors’ contributions

JJW and MN: conception and study design; interpretation of data, writing the manuscript. MN: acquisition and data analysis. JJW: obtained funding. Both authors have approved the final manuscript.
